# Composite Anion-Exchange Membrane Fabricated by UV Cross-Linking Vinyl Imidazolium Poly(Phenylene Oxide) with Polyacrylamides and Their Testing for Use in Redox Flow Batteries

**DOI:** 10.3390/membranes11060436

**Published:** 2021-06-10

**Authors:** Martyna Charyton, Cristina Iojoiu, Peter Fischer, Gerard Henrion, Mathieu Etienne, Mateusz L. Donten

**Affiliations:** 1 Amer-sil S.A., 61 Rue d’Olm, 8281 Kehlen, Luxembourg; martyna.charyton@amer-sil.com; 2 Department of Chemistry and Physics of Solids and Surfaces, Université de Lorraine, CNRS, IJL, F-54000 Nancy, France; gerard.henrion@univ-lorraine.fr; 3 Laboratoire de Chimie Physique et Microbiologie pour les Matériaux et l’Environnement, CNRS, Université de Lorraine, F-54000 Nancy, France; 4 Univ. Grenoble Alpes, Univ. Savoie Mont Blanc, CNRS, Grenoble INP, LEPMI, F-38 000 Grenoble, France; cristina.iojoiu@lepmi.grenoble-inp.fr; 5 Applied Electrochemistry, Fraunhofer Institute for Chemical Technology ICT, Joseph-von-Fraunhofer, Straße 7, 76327 Pfinztal, Germany; peter.fischer@ict.fraunhofer.de

**Keywords:** poly(phenylene oxide), coating, composite ion exchange membrane, UV curing, redox flow batteries

## Abstract

Composite anion-exchange membranes (AEMs) consisting of a porous substrate and a vinyl imidazolium poly(phenylene oxide) (VIMPPO)/acrylamide copolymer layer were fabricated in a straightforward process, for use in redox flow batteries. The porous substrate was coated with a mixture of VIMPPO and acrylamide monomers, then subsequently exposed to UV irradiation, in order to obtain a radically cured ion-exchange coating. Combining VIMPPO with low-value reagents allowed to significantly reduce the amount of synthesized ionomer used to fabricate the mem- brane down to 15%. Varying the VIMPPO content also allowed tuning the ionic transport properties of the resulting AEM. A series of membranes with different VIMPPO/acrylamides ratios were prepared to assess the optimal composition by studying the changes of membranes properties—water uptake, area resistivity, permeability, and chemical stability. Characterization of the membranes was followed by cycling experiments in a vanadium RFB (VRFB) cell. Among three composite membranes, the one with VIMPPO 15% *w*/*w*—reached the highest energy efficiency (75.1%) matching the performance of commercial ion-exchange membranes (IEMs) used in VRFBs (Nafion^®^ N 115: 75.0% and Fumasep^®^ FAP 450: 73.0%). These results showed that the proposed composite AEM, fabricated in an industrially oriented process, could be considered to be a lower-cost alternative to the benchmarked IEMs.

## 1. Introduction

Ion-exchange membranes (IEMs) are indispensable materials for a large portfolio of technologies spanning from water treatment to energy storage, for which electro-membrane processes are employed. In the context of this paper, IEMs are seen as one of the key materials in energy storage devices, such as reverse electrodialyzers over fuel cells [1,2], and in particular, redox flow batteries (RFBs) [3,4]. Beyond energy storage, IEMs find application in separation processes, such as water desalination, metal recovery or deacidification by dialysis [5], and electrodialysis [6].

In RFBs, the energy is stored in fluid materials (liquid or gas form), similar to fuel cells. The membrane that separates the anolyte and catholyte needs to prevent their mixing in convective and diffusive processes to avoid self-discharge of the electrochemical cell, seen as an energy loss [7]. Moreover, in the case of asymmetric RFBs (such as most organic systems [8] and many employing redox couples of two metals, for example, Fe–Cr [9]), the cross-mixing of active masses causes irreversible contamination of the electrolytes. While separating the redox-active species, the IEM inside of the RFB is expected to conduct common, balancing ions (often protons or chlorides [10,11]). High ion-conductivity of the membrane is necessary to minimize ohmic losses in the battery [12]. Further, the membrane has to exhibit good mechanical and chemical stability in the chosen RFB system. The electrolyte can be highly acidic [13], neutral [14,15], or (more seldom) alkaline [3]. In all cases, the membrane is exposed to oxidative media of charged electrolyte.

The most commonly used IEMs in the RFB application are the commercially available Nafion^®^ membranes fabricated out of perfluorosulfonic acid (PFSA) polymers [16]. These ionomers exhibit outstanding chemical stability, and excellent proton conductivity and ion selectivity [17,18]. However, the high cost of Nafion^®^ membranes significantly impedes their commercial utilization in RFBs [19,20]. To overcome this problem, alternative cation-exchange membranes (CEMs) based on sulfonated, non-fluorinated polymers have been developed. Their list includes sulfonated polyimides, poly(arylene ether)s, polyethersulfones, and polysulfones [21].

Apart from CEMs, anion-exchange membranes (AEMs) are reported as suitable membranes for those RFB systems that use cationic redox-active species (for example, all-vanadium redox flow batteries, VRFB [22]). Following the Donnan exclusion mechanism, the equilibrium concentration of cations in the membrane would be significantly diminished as a result of the electrostatic repulsion caused by cationic groups present in the AEM. To obtain chemically and mechanically stable, non-perfluorinated AEM, aromatic backbone polymers are preferred. Published work identified poly(phenyl sulfone) poly(aryl ether ketone), poly(benzimidazolium), or poly(phenylene oxide) (PPO) as some of the best candidates [23,24,25]. In particular, PPO has received significant interest as an excellent material for IEMs, owing to their hydrolytic stability and versatile functionalization chemistries [26] in which cationic moieties can be attached to the PPO backbone [27]. Quaternary ammonium, pyridinium, or imidazolium PPO-based AEMs have been reported as well-performing membranes in various energy storage systems [28,29,30].

The vast majority of state-of-the-art AEMs are in a form of dense, self-supported films. They are commonly made of a single ionomer designed to balance the hydrophilic and hydrophobic interactions for a compromise between material properties—tensile strength, chemical stability, and ion transport—conductivity and selectivity [31,32]. As a result, their fabrication process often requires multi-step synthesis and solvent intense processing, contributing to the high final cost of the membrane and problematic industrial scale-up. Thus, other solutions have to be investigated.

Composite membranes could be a powerful alternative. They can be either—pore-filled [33,34] or coated [35,36], obtained by modifying a porous substrate with an ionomer—in its bulk and on its surface, respectively. In both cases, the use of the porous substrate has the advantage of decoupling the materials selected for mechanical integrity and for ionic selectivity [35]. The substrate can serve as a reinforcement of the membrane; hence, the range of applicable ionomers can be extended to those that do not form dimensionally stable films.

To explore further opportunities offered by the composite membranes, a coated AEM was fabricated using a mixture of photocurable precursors applied on a porous substrate in a blade-coating process, and cross-linked upon UV irradiation. The fabrication methods are easily scalable as they are widely present in the coating industry, including commodity sectors such as paper production, packaging, printing, etc. [37,38] and often used in a roll-to-roll production set-up. The use of speciality chemicals (such as functionalized PPO) in the ion-exchange coating formulation was kept at a minimum. For the cost advantage, cheap reagents such as common, non-toxic acrylamide derivatives, were used as the majority components in the coating formulation. In addition, the final properties of the ion-exchange membrane could be tuned in a wide range of conductivity and selectivity, by adjusting the composition of the mixture of photoreactive precursors, without the need of repeating dedicated ionomer synthesis for each specimen. The proposed anion-exchange layers could not be fabricated as self-supported films. A porous substrate is proposed as a reinforcement and carrier of the ionomer coating. This allowed for including a large share of low-cost, highly hydrophilic materials in the formulation, which would not allow reaching the integrity and dimensional stability required for a self-standing film.

To demonstrate the applicability of the new membranes and find the best performing composition, their performance was evaluated using a laboratory vanadium redox flow battery (VRFB) cell. The vanadium electrolyte was chosen for the testing system, following the vast literature available for comparison of the results [39,40,41,42]. The prepared membranes were compared with benchmarked IEMs, such as Nafion^®^ N 115 and Fumsep^®^ FAP 450. The formulation of the coating for the composite hierarchical membranes was optimized until the resulting membrane reached performances comparable with these references, in terms of the VRFB cell efficiency.

## 2. Materials and Methods

### 2.1. Chemicals

*N*-[3-(dimethylamino)propyl]methacrylamide (DMEA), *N*-vinyl imidazole, *N*-hydroxyethyl acrylamide (HEAA), poly(acrylic acid) (PAA, average molecular weight 1250 kDa), *N*-bromosuccinimide (NBS), biphenyl peroxide (BPO), chlorobenzene (PhCl), and *N*,*N*-dimethylacetamide (DMAc) were purchased from Merck (Sigma Aldrich Chemical Co). IGM Resins B.V. (Waalwijk, The Netherlands) provided the 2-Hydroxy-2-methyl-1-phenylpropanone (Omnirad 1173). The pristine PPO (20 kDa, polydyspersity~2.5) was purchased from PolySciences, Inc (Warrington, PA, USA). All chemical reagents were chemically of a pure grade and were used as supplied, without further purification. Nafion^®^ 115 (N115) (Chemours, Wilmington, DE, USA) and Fumasep^®^ FAP 450 (Fumatech GmbH, Bietigheim-Bissingen, Germany) were purchased at Fuel Cell Store Inc. (College Station, TX, USA). PVC-silica porous substrates (median pore size 60 nm, volume porosity: 60%, 600 µm thick) were produced in-house by Amer-sil S.A., Kehlen, Luxembourg [43].

### 2.2. Synthesis of Vinyl Imidazolium Poly(Phenylene Oxide)

#### 2.2.1. Bromination of Poly(Phenylene Oxide)

The bromination reaction was adapted from the protocol reported by Willdorf-Cohen, Sapir et al.[44]. PPO (15.0 g, 125 mmol structural units) was slowly stirred and heated up to 136 °C in 300 mL of chlorobenzene, until it was dissolved. Then, BPO (1.6 g, 6.6 mmol) and NBS (16.7 g, 94 mmol) were added to the solution, which was stirred at reflux for another 5 h. After that, the reaction mixture was chilled to room temperature. The product was precipitated by pouring it into a 5-fold excess of methanol and stirred for 24 h. The brominated PPO (Br-PPO) was filtered and washed several times with methanol. To eliminate the residual impurities, the polymer was reprecipitated from chlorobenzene/methanol. The product in the form of white powder was dried under vacuum at 50 °C for 24 h. ^1^H NMR spectroscopy (Bruker Avance 400 spectrometer) confirmed 50% degree of bromination—(400 Hz, DMSO), δ (ppm): 2.09 (s, 9H), 4.34 (s, 2H), 6.47–6.452 (d, 2H), 6.67–6.71 (m, 2H) (Figure S1 in Supplementary Materials). The yield of the reaction was 92%.

#### 2.2.2. Synthesis of Vinyl Imidazole Functionalized PPO (VIMPPO)

***N***-vinyl imidazole (10.2 mL, 112 mmol) was added dropwise to the solution of Br-PPO (12 g, ca. 37 mmol of brominated structural units) in 300 mL of *N*,N-dimethylacetamide. The reaction was stirred at 30 °C for 48 h. The product was then precipitated by pouring into a 5-fold excess of diethyl ether and stirred overnight. The polymer was recovered by filtration and washed several times with diethyl ether. The powder was dried in vacuum, for 24 h at 30 °C. The substitution of the –CH_2_Br groups with *N*-vinyl imidazole was complete, as proven by the ^1^H NMR spectrum: (400 Hz, DMSO), δ (ppm): 2.06 (m, 9H), 5.37(m, 3H), 5.91 (t, 1H), 6.47 (d, 2H), 6.72 (s, 1H), 6.93 (m, 1H), 7.22–7.33 (m, 2H), 7.77–7.98 (m, 1H), 8.11–8.18 (m, 1H) (Figure S2 in Supplementary Materials). The yield of the reaction reached 88%.

### 2.3. Fabrication of the Composite Anion-Exchange Membrane

**Figure 1:**
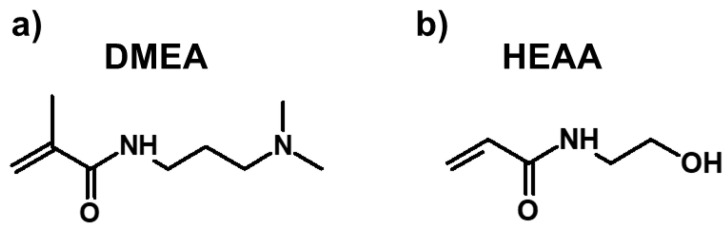
Chemical structure of the off-shelf available acrylamide monomers used for fabricating the ion-exchange coatings—(**a**) *N*-[3-(dimethylamino)propyl]methacrylamide (DMEA), (**b**) *N*-hydroxyethyl acrylamide (HEAA).

PAA (1% *w*/*w*) serving as a rheology modifier was added to the solution of DMEA and HEAA–two industrially available, safe, and non-toxic acrylamide monomers (chemical structures presented in Figure 1).

The mixture was sonicated at 45 °C for 2 h until the polymer was completely dissolved, resulting in a transparent, highly viscous solution. VIMPPO dissolved in 2 mL of methanol (viscous, brown liquid) was added, and the mixture was mechanically stirred for 10 min to obtain a homogeneous solution. Finally, the photoinitiator (Omnirad 1773, 2% *w*/*w*) was added and the mixture was stirred for another 5 min at room temperature. The masses of compounds used in each formulation are presented in Table 1. The obtained solution was applied onto the porous substrate, using a blade applicator (BYK GmbH, Wesel, Germany), with a gap of 60 µm.

A hierarchal membrane with a top anion exchange layer formed of the UV-cured ionomer alone (sample VIMPPO_100%) was fabricated to compare the properties of VIMPPO/acrylamide copolymer with a neat VIMPPO.

Radical polymerization and cross-linking of the formulations applied on the substrate were carried out under UV irradiation in a conveyor, 2-kW iron-doped mercury lamp (365 nm) (Jenton, UK).

### 2.4. Morphology Characterization

Surface and cross-section morphologies of the fabricated membranes were investigated using a scanning electron microscopy SEM (JSM IT500HR, JEOL, Tokyo, Japan) coupled with energy dispersive spectroscopy (EDS, SDD Ultim Max 170 Standard, Oxford Instruments, Abingdon, UK; software–AzrecLive). Energy dispersive spectroscopy was used to assess the elemental composition of the coating surface and to examine the cross-section of the composite membranes, with particular focus on the interphase between the anion-exchange coating and the porous support. To avoid deformation of the membranes during fracture, the samples were cut in in liquid and coated with approximately 5 nm of Au on their surface.

### 2.5. Water Uptake (WU), Area Specific Resistance (ASR), and Permeability to VO^2+^

The water uptake (*WU*) of the anion-exchange layers was determined as previously reported [24,45], according to Equation (1):

(1) $$WU=\frac{W_{W}-W_{D}}{W_{D}}\times 100\%$$

where *W_W_* and *W_D_* represent the mass of the layer under wet and dry conditions, respectively. It should be noted that, for this test, anion-exchange layers were coated on PTFE sheets and UV-cured without a porous substrate, to avoid the contribution of the substrate to the water sorption. Freshly prepared anion-exchange films were immersed in deionized water for 24 h. Before measurement, the excess water on the film’s surface was wiped off using absorbent paper. 

The area-specific resistance (ASR) of the membranes was measured in a standard 1 M HCl aqueous solution. The acidic environment was selected for consistency, with the later use of the membrane in acidic VRFB electrolyte. The difference between sulfuric and hydrochloric acid conductivity may manifest in exact values of ASR but is not expected to impact the classification of membranes from most to least conductive. The current was driven from 0 to 1 A (polarization curves), while recording the cross-membrane voltage (scan rate 10 mA s^−1^) using an Origaflex 05A (Origalys SAS, Rillieux-la-Pape, France) potentiostat. The active surface area (*A*) of the membrane was 19.63 cm^2^. Before the test, membranes were equilibrated in the electrolyte solution for 24 h. The ASR of the membrane was determined as the difference between the cell’s resistance, with and without the membrane (*R_with membrane_*, *R_without membrane_*), following Equation (2):

(2) $$\mathrm{ASR}=\left(R_{with\;membrane}-R_{without\;membrane}\right)\times A$$

In VRFB, one of the key roles of the IEM is to prevent the crossover of vanadium species, such as VO^2+^, VO_2_^+^, V^2+^, and V^3+^, between the positive and negative electrolyte. In literature, VO^2+^ is commonly used as a model, stable vanadium cation allowing to assess the permeability of the membrane in an ex-situ experiment, and thus, gain an indication of the membrane performance in terms of self-discharge and coulombic efficiency, in later RFB cell operations [45,46].

The permeability of vanadium ions (VO^2+^) across the membrane was observed using a diffusion cell. The cell was divided into two compartments (half cells) by the membrane (active area 19.63 cm^2^). One of the half-cells (feeding) was filled with 200 mL of 0.15 M vanadyl sulphate (VOSO^4^) in 3 M H_2_SO_4_. The other half-cell (receiving) contained 200 mL of 0.15 M magnesium sulphate (MgSO_4_) in 3 M H_2_SO_4_, to compensate for osmotic pressure. The number of vanadium ions (VO^2+^) that permeated across the membrane into the receiving solution was monitored over time, by recording the UV-vis spectra (Thermo Scientific™ Evolution 60S UV-visible spectrophotometer).

### 2.6. Ex-Situ Chemical Stability in VO_2_^+^

The chemical stability of the membrane was evaluated by immersing a specimen (3 cm by 0.5 cm, mass of the samples: VIMPPO_10% 0.076, VIMPPO_15% 0.081 g, VIMPPO_20% 0.079 g) in 8 mL of solution of vanadium (VO_2_^+^), in sulfuric acid, at room temperature. A commercial vanadium electrolyte (Gesellschaft für Elektrometallurgie GmbH, Nürnberg, Germany, containing 0.8 M VOSO_4_/0.8 M V_2_(SO_4_)_3_ in 2 M H_2_SO_4_ and 0.05 M H_3_PO_4_) was used to obtain the solution of VO_2_^+^, by charging it in the VRFB cell to 1.70 V and holding the voltage until the current dropped down to 10 mA. After this, the catholyte was used as a source of oxidative species for testing the stability of the membranes. Before the immersion, the membranes were washed in deionized water to remove the possibly remaining unreacted initiator or solvent. The VO^2+^ cations resulting from VO_2_^+^ reduction accompanying membrane oxidative degradation were detected using UV-vis spectroscopy. After the chemical stability tests, the cross-sections of the membranes were observed under SEM.

### 2.7. VRFB Single-Cell Performance

For cycling performance testing, the VRFB single cell was assembled with two pieces of thermally activated carbon felt electrodes (48 h at 400 °C, 50 mm × 40 mm × 4.6 mm). A total of 60 mL of commercial electrolyte was used for the anolyte and the same volume was used for the catholyte (120 mL in total, vanadium electrolyte solution, Gesellschaft für Elektrometallurgie GmbH, Nürnberg, Germany, containing 0.8 M VOSO_4_/0.8 M V_2_(SO_4_)_3_ in 2 M H_2_SO_4_ and 0.05 M H_3_PO_4_). These were circulated at a flow rate of 40 mL min^−1^. The negative electrolyte was kept under nitrogen to prevent undesired oxidation of V^2+^. The cycling tests were carried out using Origalys potentiostat, at constant current densities of 20 mA cm^−2^, 50 mA cm^−2^, and 80 mA cm^−2^. The upper and lower limits of the charge/discharge voltage were set at 1.75 and 0.80 V, respectively. The coulombic efficiency (CE), energy efficiency (EE), and voltage efficiency (VE) of the membrane were calculated, based on Equations (3)–(5):

(3) $$\mathrm{CE}\left(\%\right)=\frac{\int^{\;}I_{d}\;dt}{\int^{\;}I_{c}\;dt}\times 100\;\%$$

(4) $$\mathrm{EE}\left(\%\right)=\frac{\int^{\;}V_{d}\;I_{d}\;dt}{\int^{\;}V_{c}I_{c}\;dt}\times 100\;\%$$

(5) $$\mathrm{VE}\left(\%\right)=\frac{\mathrm{EE}}{\mathrm{CE}}\times 100\;\%$$

where *I_d_* and *I_c_* stand for the discharging and charging current, respectively, while *V_d_* and *V_c_* are discharging and charging voltages.

To observe the crossover of the vanadium species in an RFB device, open-circuit voltage (OCV) of the charged VRFB cell (after a complete charge up to 1.7 V) was observed, until the voltage of the cell decreased below 0.8 V. The cell was filled with a total amount of 20 mL of vanadium electrolyte, recirculated with a flow rate of 40 mL min^−1^. The volume of electrolyte was reduced, as compared to the cycling experiments, in order to limit the batteries’ electrical capacity and obtain the discharge results in a shorter period of time. The drop of the OCV of the cell was directly related to the diffusion of the vanadium ions at various oxidation states across the membrane, separating the two half-cells, and resulting in the self-discharge reactions.

## 3. Results

### 3.1. Synthesis of Vinylimidazolium Poly(Phenylene Oxide)

**Figure 2:**
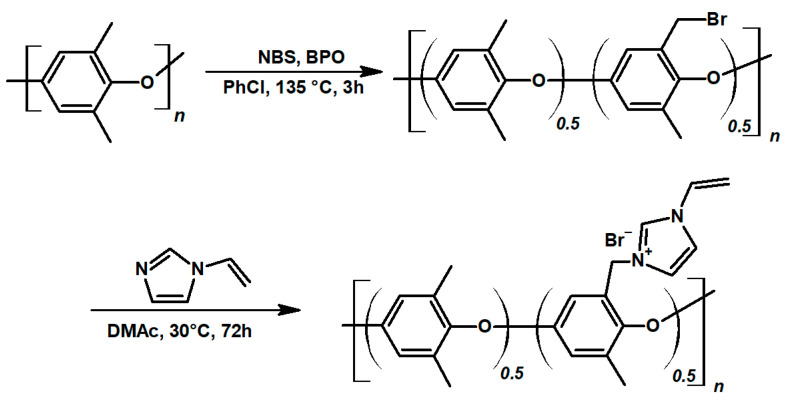
Synthetic route for obtaining the poly(phenyl oxide) quaternarized with N-vinyl imidazole (VIMPPO).

Quaternary ammonium poly(phenylene oxide) was obtained in a two-step synthesis, following the protocol described in the experimental section; presented in Figure 2. The aim was to obtain an ionomer with a high ion-exchange capacity, to guarantee excellent ionic conductivity and selectivity of the membrane. Therefore, in the first step, 50% of polymer structural units were brominated. In the quaternarization step, all benzyl bromide groups were successfully substituted with N-vinyl imidazole, resulting in an ionomer with a theoretical ion-exchange capacity of 2.4 mmol g^−1^ (H NMR data confirming the synthesis products are available in Supplementary Materials Figures S1 and S2). Crosslinked vinyl imidazolium poly(phenylene oxide) self-supported membranes were reported in the works on fuel cells [47]. In this study, N-vinyl imidazole was selected for the functionalization of PPO, due to the presence of vinyl moiety, which allows VIMPPO to react with other radically reactive monomers in the process of UV curing. Effectively, VIMPPO is used to introduce positively charged groups and act as a cross-linker for the coating. In this way, it contributes to ionic conductivity and stability of the ionomer layer. Unlike the state-of-the-art imidazolium PPO-based membranes [48,49], herein, the AEMs were obtained by diluting the VIMPPO with acrylamides, significantly diminishing the amount of needed ionomer, which could be seen as an economical benefit.

### 3.2. Membrane Fabrication and Morphology Characterization

The VIMPPO obtained in the synthesis described above reached an excessively high cross-linking degree when UV-cured alone. Such coating layers were expected to exhibit remarkably low permeability of cations, at the cost of a low overall ionic conductivity. Diminishing the content of VIMPPO in the coating to below 25% *w*/*w* by introducing acrylamide derivative monomers in the formulation, led to a decreased cross-linking density caused by the incorporation of a large fraction of hydrophilic polyacrylamide, in the resulting, cross-linked polymer. DMEA monomer has a tertiary amine group in its chemical structure. This weakly basic group (-NR_2_) possess a lone pair of electrons which becomes protonated in the acidic electrolyte of VRFB [50]. The resulting -NR_2_H^+^ moiety facilitates a labile proton, which can be transferred to another amine group participating in the Grotthus mechanism of proton hopping, contributing to the ionic conductivity [51]. Hence, the anion exchange membrane can also be considered to be a proton exchanger. Effectively, formulating VIMPPO in a mixture of acrylamide monomers enabled to control the trade-off between the cation permeability and the resistivity of the coating. The VIMPPO content ranged from 10 to 25% *w*/*w* in the fabricated coatings.

VIMPPO/acrylamide copolymers were found to be highly hydrophilic in the studied range of formulations and would not be able to form self-supported membranes. The lowest content of VIMPPO providing sufficient cross-linking for obtaining a stable coating layer was 10% *w*/*w*. The VIMPPO_10% sample reached water uptake values of 74%. During attempts to fabricate the membrane samples with VIMPPO content in the coating below 10% *w*/*w*, the coating layers were found to be unstable in aqueous electrolyte and separated from the substrate in the form of a soft gel. In such cases, crosslinking was found to be insufficient to secure the integrity of the ionomer film. 

Taking the advantage of the dimensional and mechanical stability of the porous PVC-silica substrate, the anion exchange layers were kept at a maximum of 50 µm. The final thickness of the coating layer was determined by the gap of the blade applicator, the viscosity of the applied mixture and shrinkage occurring during the radical polymerization. Varying the content of VIMPPO (10–25%) could potentially affect the viscosity and cure shrinkage of specific formulations. The fabricated anion exchange layers had an overall average thickness in a range of 20–40 µm when cured and dried. Within one sheet of the membrane, thickness deviation due to the uneven surface of the substrate were present. As a result, the thickness of the coating layer could be locally different (minimum of 15 µm and a maximum of about 50 µm were noticed in the analyzed dataset). The ununiform surface of the substrate additionally rich in the exposed grains of silica particles was found to be advantageous. Such surface provided for very good adhesion between the substrate and the polymeric coating. Other types of porous materials such as thin poly(ethylene) or poly(propylene) porous sheets (stretched foils) or cellophane membranes did not provide sufficient adhesion between the substrate and coating. When coating attempts were taken on such supports, the ionomer formulation would not distribute evenly during the coating, and despite curing, delaminate upon wetting and swelling. This was not an issue in the case of the PVC-Silica heterogeneous porous membrane used in this work. The use of thick PVC-silica substrate (0.6 mm) was not expected to cause substantial ion transport limitations, since the VRFB electrolyte is highly conductive (2 M H_2_SO_4_). In such an environment, effective current transport in electromigration is expected, even in areas inside the substrate that are free of convective transport. The same type of PVC-Silica separators were used before as porous separators in vanadium and copper RFBs [52,53], and analogue examples of PE-Silica separators were also found [54].

**Figure 3:**
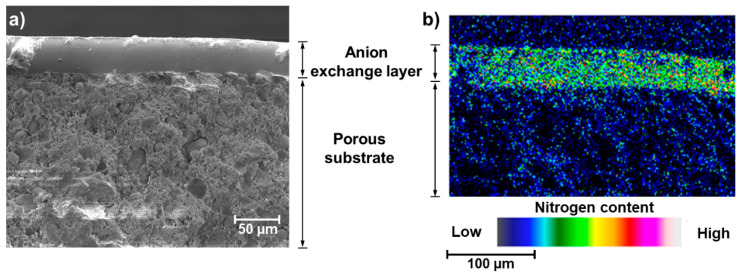
SEM/EDS micrographs showing the hierarchical structure of the coated composite membrane. (**a**) SEM cross-section image; (**b**) EDS Nitrogen map of the cross-section.

SEM images and EDS analysis confirmed a hierarchical, layered, structure of the fabricated membrane, as illustrated with the cross-sections view and elemental map in Figure 3 (SEM image of the surface and corresponding elemental map are available in Supplementary Materials Figure S3).

### 3.3. Area-Specific Resistivity (ASR) and Water Uptake (WU) of the Membranes

**Figure 4:**
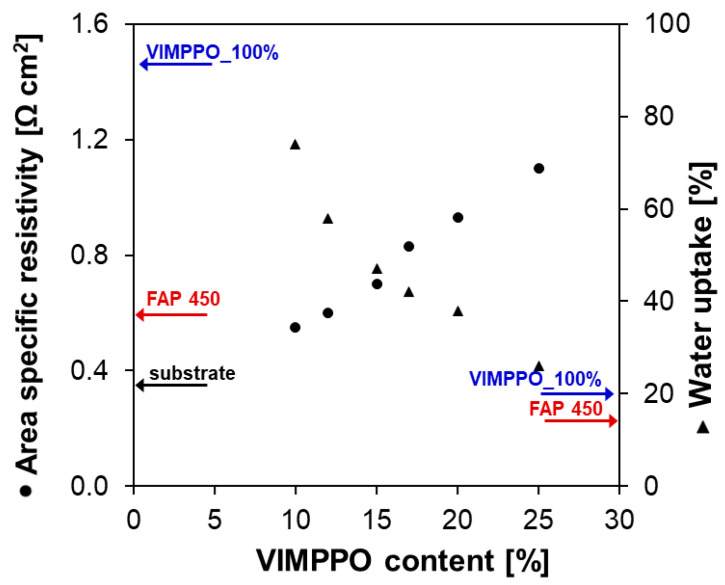
Area-specific resistance measured in 1 M HCl_aq_ at a current scan rate of 10 mA s^−1^ and water uptake of the membranes in a function of VIMPPO content in the anion-exchange coating.

Figure 4 shows how the water uptake (WU) and the area-specific resistance (ASR) were changing, depending on the content of VIMPPO in the coating layer. The VIMPPO_100% membrane exhibited the highest ASR (1.4 Ω cm^2^) and the lowest WU (20%) among the fabricated membranes, due to its densely cross-linked structure (values marked with blue arrows in Figure 4). When the content of VIMPPO was reduced and the ionomer was used as a functional crosslinker for the formulation of polyacrylamides, the ion conductivity of the membrane was significantly improved. The ASR value dropped from 1.4 Ω cm^2^ to 1.1 Ω cm^2^ at 25% *w*/*w* of VIMPPO, and further down to 0.55 Ω cm^2^ at 10% *w*/*w*.

The ASR trend following the diminishing content of VIMPPO in the coating is understandable. Formulations with the lower content of VIMPPO are expected to reach a lower density of cross-linking, allowing for a higher water uptake. The water uptake trend was also confirmed by the experimental data shown in Figure 4.

Figure 4 allows comparing the resistivity and water uptake of the composite membranes, with results obtained for FAP 450, the commercial benchmark (WU—15%, ASR—0.58 Ω cm^2^; red arrows in Figure 4).

In the case of the composite membranes, both the anion-exchange coating and the porous substrate contributed to the overall ASR of the membrane. To isolate these contributions, the ASR of the porous substrate was measured, giving 0.37 Ω cm^2^. This value could be subtracted from the ASR of the entire coated membrane reported in Figure 4 to estimate the resistivity of the coating alone. For example, the ASR value of VIMPPO_10% membrane was 0.55 Ω cm^2^, out of which only 0.18 Ω cm^2^ was related to the coating after subtracting the ASR of the substrate. This value was remarkably below the ASR of the FAP 450 membrane. Two properties of the coating layer could be seen as sources of this advantage in the ion conductivity. High water uptake of the VIMPPO_10% coating facilitated ion transport. In addition, the coating layer was only 20 µm thick, less than half of the thickness of the FAP 450 membrane (nominal thickness of 50 µm). A similar calculation could be computed for the VIMPPO_25% membrane; this coating contributed 0.73 Ω cm^2^ to the overall membrane ASR. Its water uptake was 26%, closer to that of FAP 450. The above argumentation indicates that a further decrease of the resistivity of the composite membrane could be achieved by further optimizing the porous substrate.

### 3.4. Permeability to Vanadium Ions

**Figure 5:**
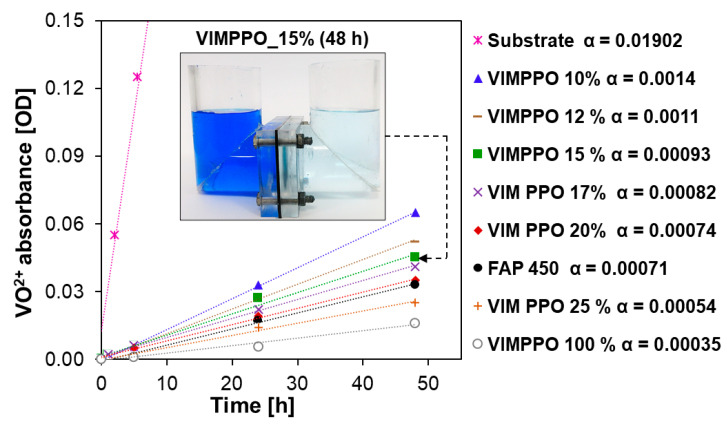
Changes in VO^2+^ absorbance (measured at 775 nm) recorded for the receiving solution over 48 h (initial solutions—receiving: 0.15 M MgSO_4_ in 3 M H_2_SO_4_, donor: 0.15 M VOSO_4_ in 3 M H_2_SO_4_). The picture inset presents the diffusion cell used for the permeability test assembled with VIMPPO_15% after 48 h of the experiment. Slopes of the absorbance curves (α) were calculated and used to quantify the permeation rate of vanadium cations.

The lowest permeability of the vanadium species (VO^2+^) among all tested membranes was observed for the neat VIMPPO coating (VIMPPO_100% sample, grey circles in Figure 5). High cross-linking density allowed neat VIMPPO to form an exceptionally effective barrier for cation transfer. When the cross-linking density was diminished by reducing the VIMPPO content in the coating to 20% *w*/*w*, the coated membrane reached vanadium permeability matching that of FAP 450. At lower contents of VIMPPO, the permeation of vanadium through the corresponding membranes increased gradually. In the case of the lowest amount of VIMPPO (10% *w*/*w*), the transfer of vanadium species through the membrane was still more than ten times slower, in comparison to a bare substrate. Slopes of the vanadium concentration in the receiving cell as function of time changed from α = 0.01902 for the substrate to α = 0.0014 for VIMPPO_10% and down to α = 0.00054 for VIMPPO_25%. Exact permeability coefficients could be calculated but because of the hierarchical structure of the coated membrane and uncertainty related to the exact uniformity and thickness of the coating layers, they are reported in the Supplementary Materials Table S3.

**Figure 6:**
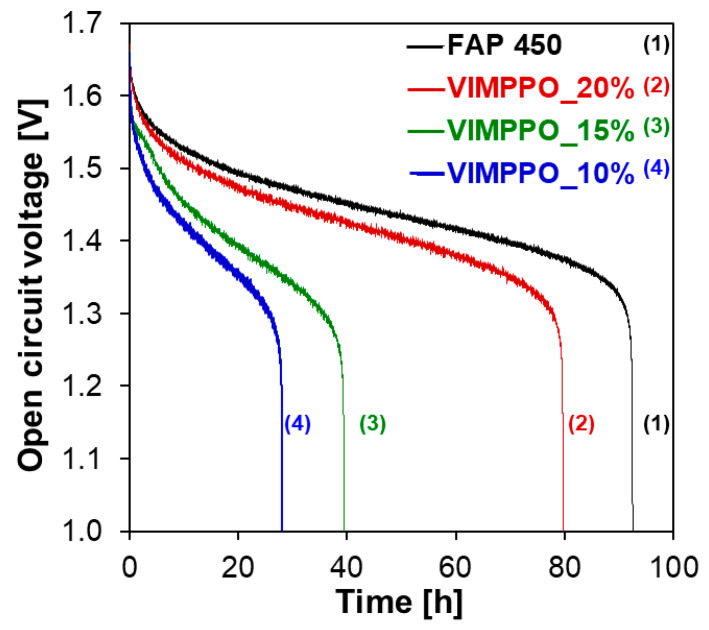
Open-circuit voltage in function of time (self-discharge curves) recorded for FAP 450 and three composite membranes—VIMPPO_20%, VIMPPO_15%, and VIMPPO_10%.

Three composite membranes—VIMPPO_10%, _15%, and _20% were chosen for further study of vanadium crossover under the VRFB cell conditions. Self-discharge curves of a cell assembled with the three listed membranes and FAP 450 were recorded after an initial, complete charge. The experiments were performed with the minimal possible volume of electrolyte (10 mL in each of the two half cells, 20 mL total), in a test cell with 20 cm^2^ of active area. The results are shown in Figure 6. The curve obtained for FAP 450 was considered to be a reference value for interpretation. Among the composite membranes, VIMPPO_20% allowed for the slowest self-discharge, the cell retained charge for 79 h. Self-discharge of the cell with FAP 450 took 92 h. For membranes with a lower content of VIMPPO in the coating layer, 39 h and 28 h were recorded for VIMPPO_15% and VIMPPO_10%, respectively. The results of the self-discharge test come in a trend qualitatively matching VO^2+^ permeability experiments. The discrepancies between the relative self-discharge and results of the vanadium permeability experiments could be explained by the migration of vanadium ions other than VO^2+^_._ Indeed, cations such as VO_2_^+^ and V^2 +^ play a significant role in the self-discharge process.

### 3.5. Ex-Situ Chemical Stability in Vanadium (V) Electrolyte

**Figure 7:**
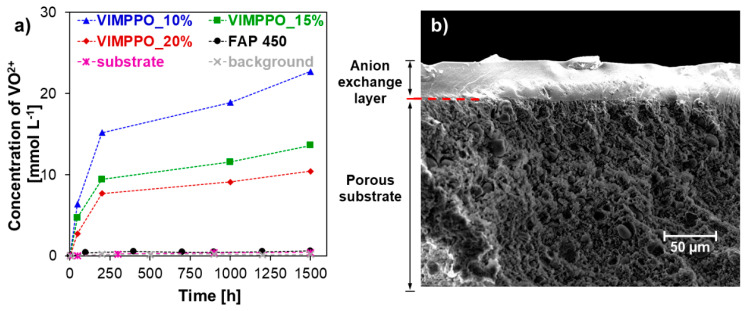
The results of oxidative stability test performed by the ageing membrane samples (VIMPPO_20%, VIMPPO_15%, VIMPPO_10%) in 1.6 M VO_2_^+^ in 2 M sulfuric acid solution. (**a**) Change in the concentration of VO^2+^ measured over 1500 h; and (**b**) SEM cross-section image of VIMPPO_15% after 1500 h of ageing.

The chemical stability in acidic and oxidative media was tested for 10, 15, and 25% *w*/*w* of VIMPPO in the coating layers. The membranes were placed in a 1.6 M solution of VO_2_^+^ in 2 M H_2_SO_4_ for over 1500 h. The results presented in Figure 7a shows that the concentration of VO^2+^ rose faster for the coatings with a lower content of VIMPPO (the UV spectra after 1500 h and 48 h are available in Supplementary Materials, Figure S4). Upon completing the 1500 h oxidation tests, the coatings were found to be intact. Figure 7b shows a cross-section image of the VIMPPO_15% membrane after the test.

The short-term stability test (48 h) conducted for individual components of the coating—cross-linked VIMPPO and polymerized acrylamides (VIMPPO free) demonstrated that no degradation occurred for VIMPPO, while oxidation took place for the polymerized acrylamides (relevant UV spectra are available in Supplementary Materials, Figure S5). The oxidation of polyacrylamide was likely to originate from the methyl and hydroxide groups present in N-[3-(dimethylamino)propyl]methacrylamide and N-hydroxyethyl acrylamide. Future formulation optimalization will focus on the replacement of these components.

Figure 7a demonstrates that the oxidation of the tested coated membranes happened mostly within the first 200 h of the test. At later times, the reactions were majorly slowed down or nearly stopped, especially in the case of the VIMPPO_15% and VIMPPO_20% membranes. The slower rate of degradation observed for the membranes with a higher amount of VIMPPO could be also attributed to the denser cross-linking. Such coatings also exhibited lower water uptake and swelled less, therefore, the attack of oxidative species could be additionally impeded by steric hindrance.

The collected information gave sufficient grounds to propose the composite membranes for VRFB testing, considering that at least the formulations with a higher content of VIMPPO would sustain the prolonged exposure to the vanadium electrolyte.

### 3.6. VRFB Single Cell Performance

The RFB cycling test aimed to find the trade-off between the opposite trends of the ASR and vanadium permeability of the membranes, which allowed for the highest energy efficiency of the RFB cell. Composite membranes with 10, 15, and 20% *w*/*w* of VIMPPO content in the coating layer were selected for this experiment; FAP 450 and Nafion^®^ N115 served as performance benchmarks [55,56,57,58,59].

**Figure 8:**
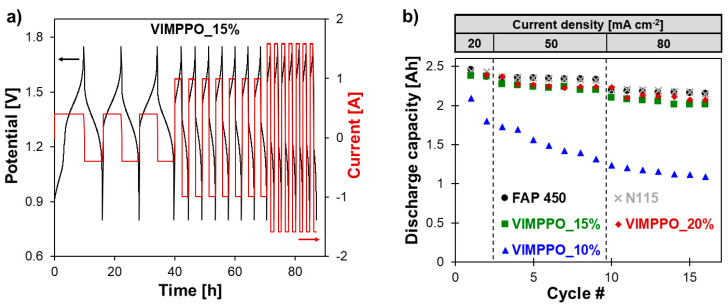
VRFB cycling performance data—(**a**) charge–discharge curve recorded for the VRFB single cell (Pinflowes, 20 cm^2^ of active area) assembled with VIMPPO_15% at three current densities—20, 50, and 80 mA cm^−2^; and (**b**) discharge capacity for each tested membrane.

The charge–discharge tests were carried out at three current densities—20, 50 and 80 mA cm^−2^. Performance at a current density exceeding 80 mA cm^−2^ was not studied, due to the RFB cell limitations, leading to a poor energy efficiency and a low effective cell capacity observed, even for the market-proven benchmarks. Exemplary charge–discharge curves recorded for the VRFB single-cell, assembled with a composite membrane, is presented in Figure 8a. The cell showed stable cycling performance at all applied current densities over the experiment duration of 5 days, repeated twice without disassembling the cell and starting with a fresh electrolyte. In another experiment, the cell was cycled exclusively at a current density of 80 mA cm^−2^ for over 50 cycles. These results are available in Supplementary Materials Figure S6. In this case, a stable operation was also recorded with energy efficiency in line with the discussion below.

Figure 8b shows cell discharge capacities of the three listed membranes and commercial IEMs extracted from the charge–discharge curves. The coating layers of the composite membrane had a decisive role in affecting the charge retention in the battery. In the case of the two composite membranes with a higher content of crosslinking ionomer (VIMPPO_20% and VIMPPO_15%), the results of capacity retention followed the evolution of data obtained for FAP 450 and N115. Using the VIMPPO_10%, membrane led to a steep loss of capacity at each applied current density. This was attributed to the significantly higher vanadium permeability through the insufficiently crosslinked structure of the VIMPPO_10% coating layer.

**Figure 9:**
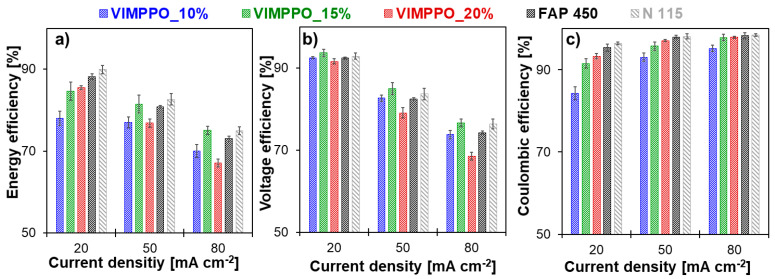
VRFB performances reached by the single-cells with the three tested composite membranes, FAP 450 and Nafion^®^ N115 cycled at 20, 50, and 80 mA cm^−2^—(**a**) energy efficiency, (**b**) voltage efficiency, and (**c**) coulombic efficiency.

Figure 9 shows the cell efficiencies (energy, voltage, and coulombic) calculated for all cycling experiments (numerical data are available in the Supplementary Materials Table S1). As is typical for RFB cells, the coulombic efficiency increased for all membranes, with increasing current densities; Figure 9c. An opposite trend was observed for voltage efficiency, Figure 9b. Loss of voltage efficiency with the increasing current density—caused by ohmic losses—dominated the trend of energy efficiency, which also diminished with increasing current density; Figure 9a. Among the coated membranes, VIMPPO_15% was seen as the best performing one, based on energy efficiency data presented in Figure 9a. At low current density (20 mA cm^−2^), its result was matched by VIMPPO_20%. The energy efficiency of VIMPPO_15% at higher current densities, closely followed the values recorded for N 115 and slightly surpassed the energy efficiency values of FAP 450 at the highest applied current (75.1% for VIMPPO_15% vs. 75.0% for N 115 vs. 73.0% for FAP 450, at 80 mA cm^−2^).

Trends observed for the energy efficiency were interpreted based on coulombic and voltage efficiencies data of the tested membranes; presented in Figure 9b,c, together with the characterization discussed earlier.

Coulombic efficiencies achieved by the cells assembled with the tested membranes (Figure 9c) was in line with the permeability data reported previously. The cell with the most densely cross-linked membrane (VIMPPO_20%), reached the highest coulombic efficiency among the other tested composite membranes (97.1% and 97.9% at 50 and 80 mA cm^−2^, respectively). The high coulombic efficiency was achieved at the cost of low voltage efficiency, caused by the highest ASR of VIMPPO_20% membranes. Therefore, the overall performance of the VIMPPO_20% membrane was suboptimal, particularly, at higher current densities conditions, in which ohmic losses dominated the cell performance (energy efficiency—76.8% and 67.1% at 50 and 80 mA cm^−2^, respectively). Diminishing the VIMPPO content in the coating layer led to a decreased coulombic efficiency—93.3% for VIMPPO_20%, 91.5% for VIMPPO_15%, and 84.5% for VIMPPO_10% recorded at 20 mA cm^−2^—current conditions at which coulombic losses were most pronounced. Therefore, higher permeation of vanadium species across the membrane was observed in the case of the less densely cross-linked membranes. This observation was in line with the permeability and self-discharge experiments.

The voltage efficiency trend of the three composite membranes was found in partial contrast to the ASR data reported earlier. ASR results correlated accurately to the content of VIMPPO in the coatings. However, despite the lowest resistivity, VIMPPO_10% did not allow us to reach proportionally high-voltage efficiencies during the RFB cycling experiments. This discrepancy is not completely understood but may have been caused by different conditions during the two experiments. ASR tests were performed in 1 M HCl with a short-lasting measurement that possibly did not include all concentration polarization effects that could build up over time, during the charge–discharge cycle in 2 M H_2_SO_4_. For the other two coated membranes, the results of voltage efficiency were in line with the ASR data. Undisputedly, the cell with VIMPPO_20% reached the lowest voltage efficiencies at all applied current densities (91.7%, 79.1%, and 68.5% for 20, 50, and 80 mA cm^−2^), resulting in a compromised energy efficiency, particularly at higher current densities. No transport limitations related to the porous substrate were manifested during the cycling experiments. The composite membranes—VIMPPO 15% and VIMPPO 10% reached voltage efficiencies comparable to the ones of the think foil commercial IEMs. Thus, it can be concluded that the ionic transport properties of the composite membranes are controlled by the ionomer coating layer. 

The cell assembled with the middle option membrane, VIMPPO_15%, reached the highest voltage efficiency values, given the unsatisfactory low voltage efficiency of VIMPPO_10%. Further it exhibited coulombic efficiencies in between the results of VIMPPO_10% and VIMPPO_20% membranes. When coulombic and voltage efficiency are combined into energy efficiency, VIMPPO_15% is considered to be the overall best performing membrane with comparable performance to the N 115 membrane, at the highest current density.

## 4. Conclusions

The proposed composite hierarchical anion-exchange membranes were able to reach the low, area-specific resistance, and low cation (VO^2+^) permeability, when formulated for the right level of cross-linking ionomer (VIMPPO). Compiling the characterization and RFB cell cycling results, VIMPPO_15% exhibited the optimal trade-off between the various properties determining the performance of a membrane in a VRFB cell. With 75.1% of energy efficiency at current density 80 mA cm^−2^, the VIMPPO_15% membrane closely matched the performance of the PFSA-based benchmark cation-exchange membrane N 115 (75.0%) and exceeded the performance of a commercial anion-exchange membrane FAP 450 (73.0%). This was seen as an achievement, given that the coated membrane was fabricated in a straightforward manner and was utilized in the majority (85% *w*/*w*), low-value materials. Acrylamide monomers are readily available at the chemical reagent market and can be replaced with industrial commodity chemicals—DMAPAA^TM^ or HEAA^TM^ (KJ Chemicals Corporation, Tokyo, Japan). The proposed fabrication method, employing blade coating and UV curing steps, opens direct scale-up possibilities.

Despite the use of non-perfluorinated compounds, the chemical stability of the composite membranes was sufficient for their investigation. Indication of oxidation of the polymerized acrylamide was observed during the VO_2_^+^ ageing tests but in none of the experiments did the membrane display visible signs of degradation. Experimental data showed that other components of the formulation were less resistant to V(V) than the VIMPPO polymer itself. This aspect will require future attention and possible improvements of the membrane formulation.

This study shows that the ionic transport properties of the composite membrane can be conveniently controlled by changing the amount of VIMPPO used for the fabrication of the ion-exchange coating layer. Decreasing the content of VIMPPO in the coating, resulted in a lower density of cross-linking, and thus, higher water uptake, lower ASR, and higher cation permeability. In this way, the balance between coulombic efficiency and voltage efficiency of the cell could be controlled.

In conclusion, the presented hierarchical membrane is a promising concept for low-cost IEMs that may be used in energy storage applications.

## Figures and Tables

**Table 1:** The compositions of the formulations used for fabricating the ion-exchange coatings.

	VIMPPO [g]	DMEA [g]	HEAA [g]	PAA [g]	Initiator [g]
VIMPPO_10%	0.7	3.5	2.5	0.08	0.15
VIMPPO_12%	0.85	3.5	2.5	0.08	0.15
VIMPPO_15%	1.1	3.5	2.5	0.08	0.15
VIMPPO_17%	1.3	3.5	2.5	0.08	0.15
VIMPPO_20%	1.6	3.5	2.5	0.08	0.15
VIMPPO_25%	2.1	3.5	2.5	0.08	0.15
VIMPPO_100%	2.5	0	0	0	0.05

## Data Availability

Inquiries regarding data used in the paper can be addressed to the corresponding authors. All data needed for motivating the conclusions of the paper are available in the manuscript, additional background information can be found in the Supplementary Materials.

## Supplementary Materials

The following are available online at https://www.mdpi.com/article/10.3390/membranes11060436/s1. Figure S1. ^1^H NMR spectrum of the brominated PPO (Bruker Avance 400 spectrometer). Figure S2. ^1^H NMR Spectra of vinylimidazolium PPO (VIMPPO) (Bruker Avance 400 spectrometer). Figure S3. SEM image of ion-exchange coating surface (left), EDS map—nitrogen distribution (right). Figure S4. UV-vis spectra recorded for the tested solution (ex-situ chemical stability) comparison between the different composite membranes. Figure S5. UV–Vis spectra recorded for the tested solution (ex-situ chemical stability—short-term stability of the coating’s component; matrix UV-cured acrylamides), and VIMPPO-cured alone. Figure S6. Cycling performance of the VRFB cell assembled with the membrane VIMPPO_15% over 50 cycles at 80 mA cm^−2^—(a) charge–discharge curves recorded, (b) coulombic, voltage, and energy efficiency of the cell. Table S1. Performance of VRFB single cells (active area, 20 cm^2^) assembled with different membranes. Table S2. Permeability coefficients calculated for the composite membranes and the commercial AEM–FAP 450.
