# Supplementary material for: Composite Anion-Exchange Membrane Fabricated by UV Cross-Linking Vinyl Imidazolium Poly(Phenylene Oxide) with Polyacrylamides and Their Testing for Use in Redox Flow Batteries
Source: Membranes (Basel). 2021 Jun 10;11(6):436. doi: 10.3390/membranes11060436 (PMC8227260; doi:10.3390/membranes11060436)
Supplement: Supplementary file 1 [file membranes-11-00436-s001.zip › membranes-1227753-supplementary.pdf]

membranes

Supplementary File

Composite Anion-Exchange Membrane Fabricated by UV Cross-Linking Vinyl Imidazolium Poly(Phenylene Oxide) with Polyacrylamides and Their Testing for Use in Redox Flow Batteries

Martyna Charyton [1,2,3], Cristina Iojoiu [4], Peter Fischer [5], Gerard Henrion [2], Mathieu Etienne [3,*] and Mateusz L. Donten [1,*]

[1] Amer-sil S.A., 61 Rue d'Olm, 8281 Kehlen, Luxembourg; martyna.charyton@amer-sil.com
[2] Department of Chemistry and Physics of Solids and Surfaces, Université de Lorraine, CNRS, IJL, F-54000 Nancy, France; gerard.henrion@univ-lorraine.fr
[3] Laboratoire de Chimie Physique et Microbiologie pour les Matériaux et l'Environnement, CNRS, Université de Lorraine, F-54000 Nancy, France
[4] Univ. Grenoble Alpes, Univ. Savoie Mont Blanc, CNRS, Grenoble INP, LEPMI, F-38 000 Grenoble, France; cristina.iojoiu@lepmi.grenoble-inp.fr
[5] Applied Electrochemistry, Fraunhofer Institute for Chemical Technology ICT, Joseph-von-Fraunhofer, Straße 7, 76327 Pfinztal, Germany; peter.fischer@ict.fraunhofer.de
* Correspondence: mathieu.etienne@univ-lorraine.fr (M.E.); mateusz.donten@amer-sil.com (M.L.D.)

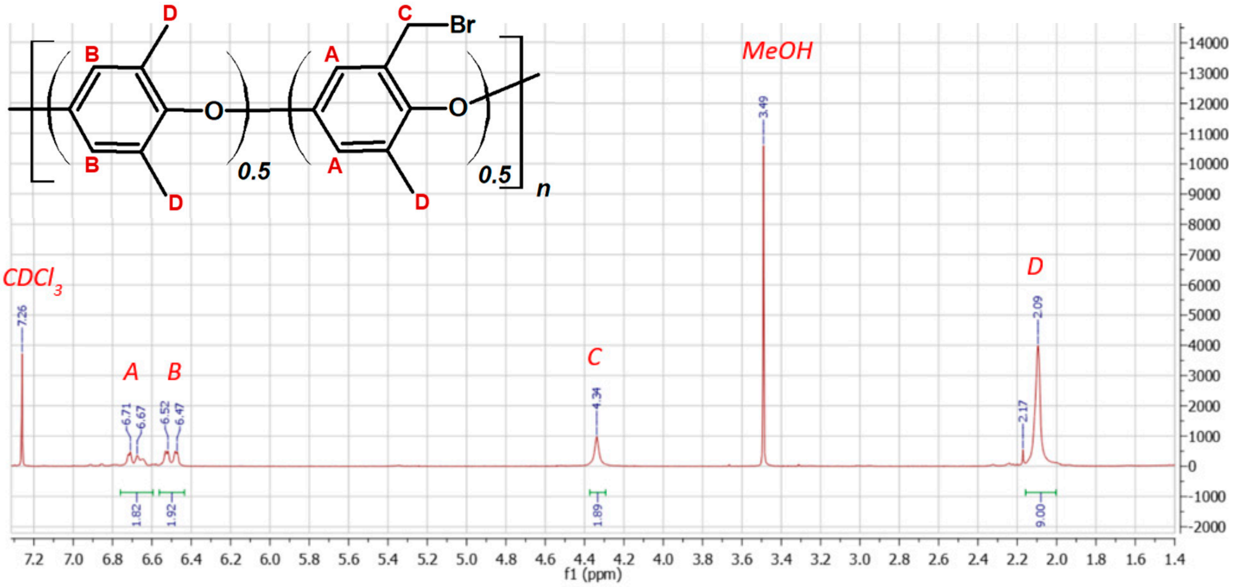

Figure S1. ^{1}H NMR spectrum of brominated PPO (Bruker Avance 400 spectrometer).

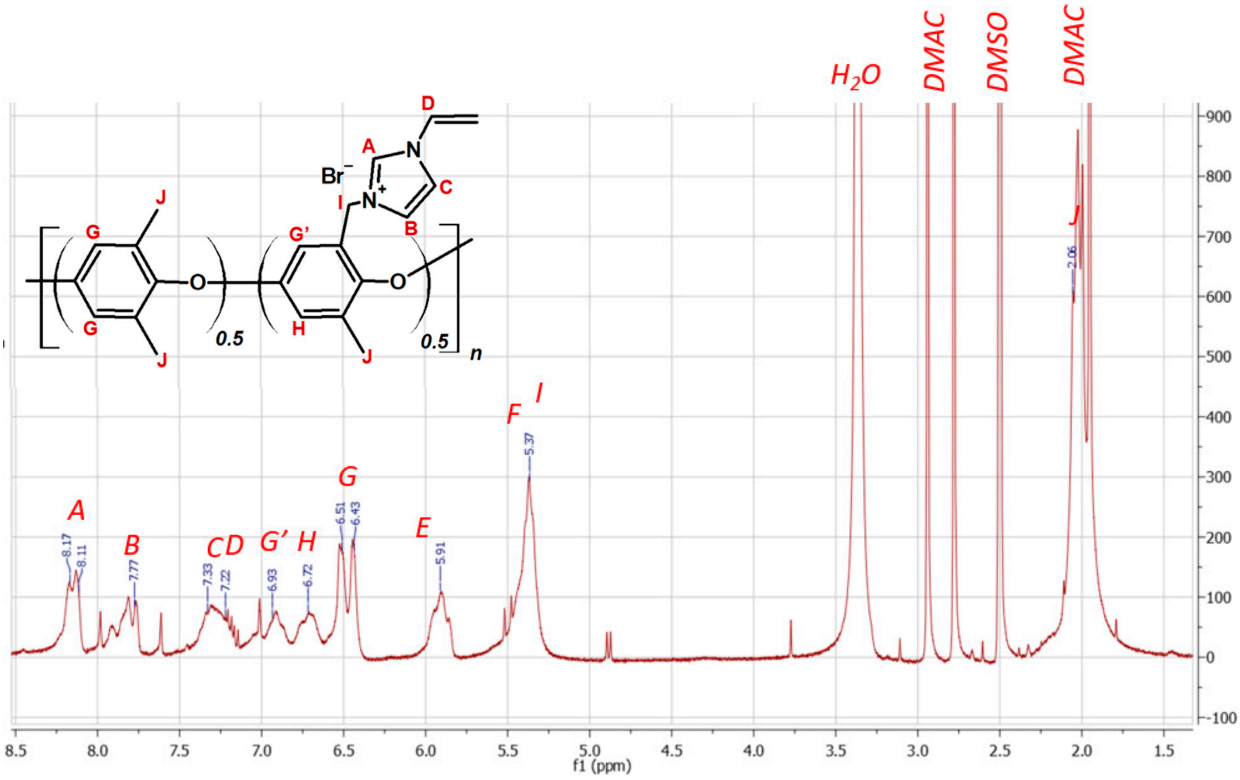

Figure S2. ^{1}H NMR Spectra of vinylimidazolium PPO (VIMPPO) (Bruker Avance 400 spectrometer).

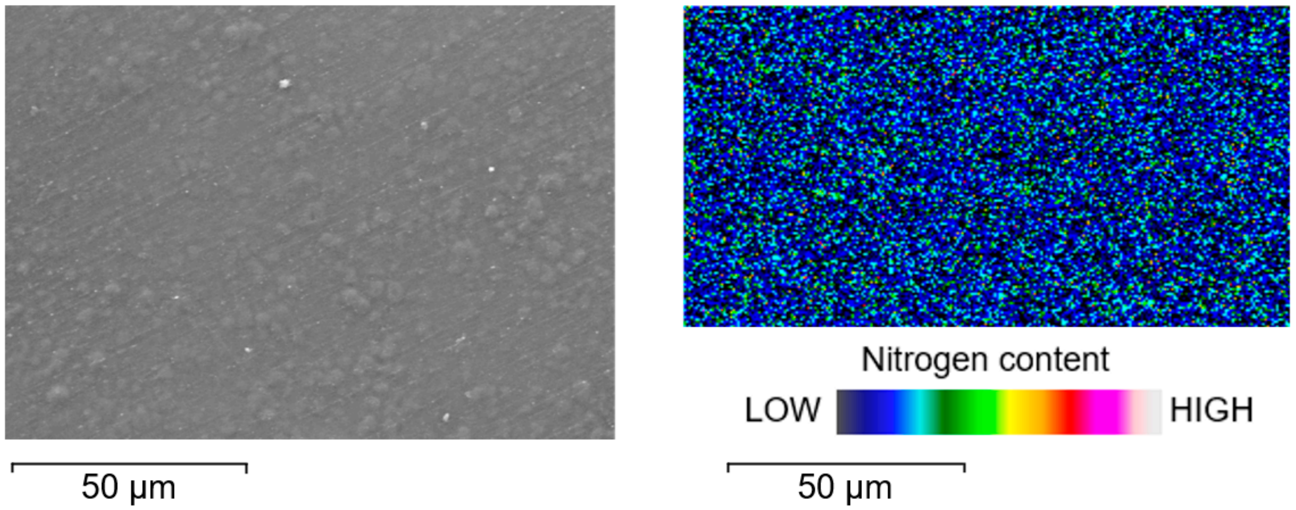

Figure S3. SEM image of ion exchange coating surface (left), EDS map - Nitrogen distribution (right).

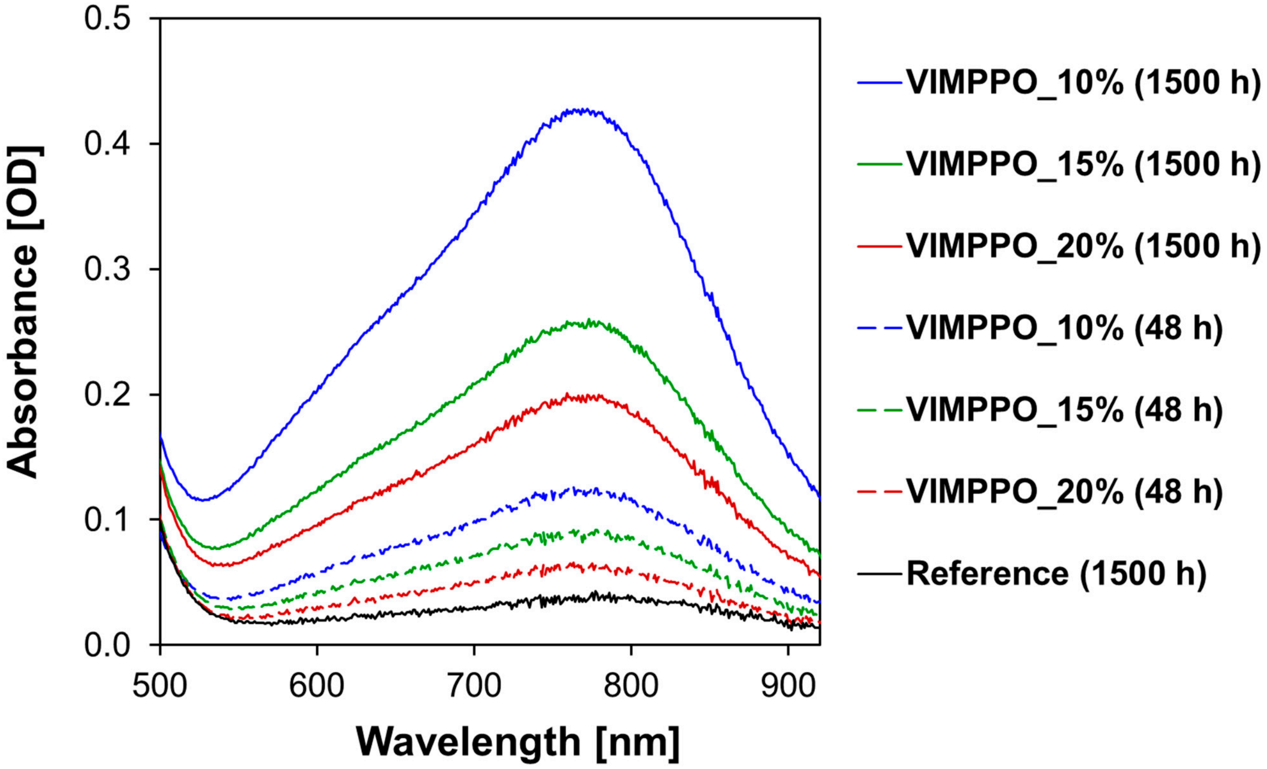

Figure S4. UV-vis spectra recorded for the tested solution (Ex-situ chemical stability) - comparison between the of different composite membranes.

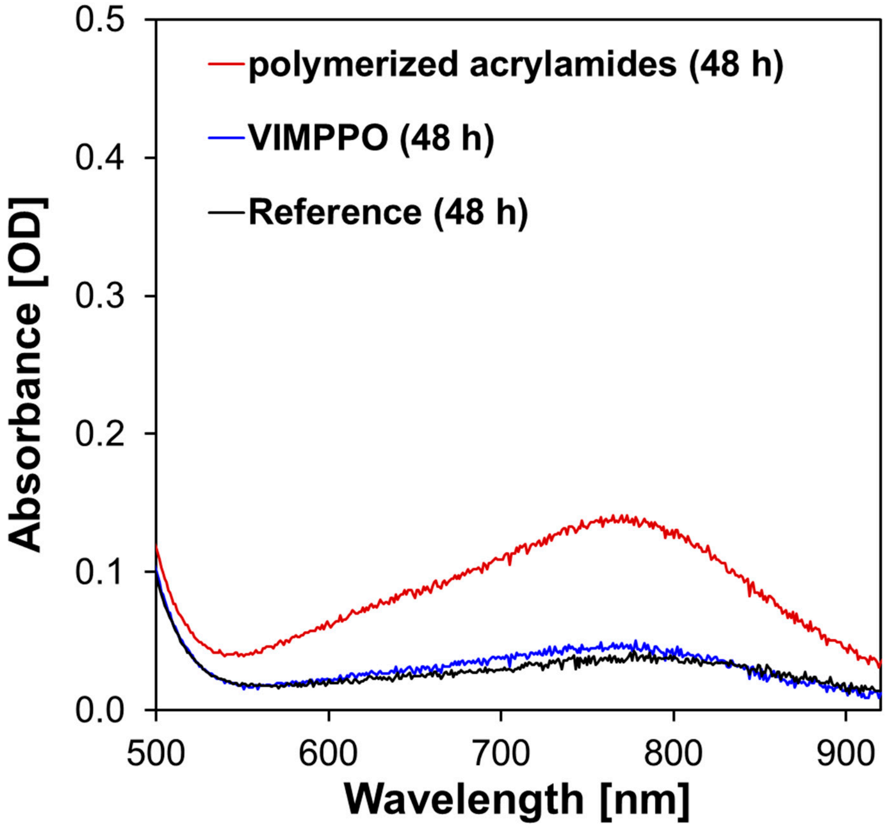

Figure 5. UV-vis spectra recorded for the tested solution (Ex-situ chemical stability: short term stability of the coating's component: matrix UV-cured acrylamides), and VIMPPO cured alone.

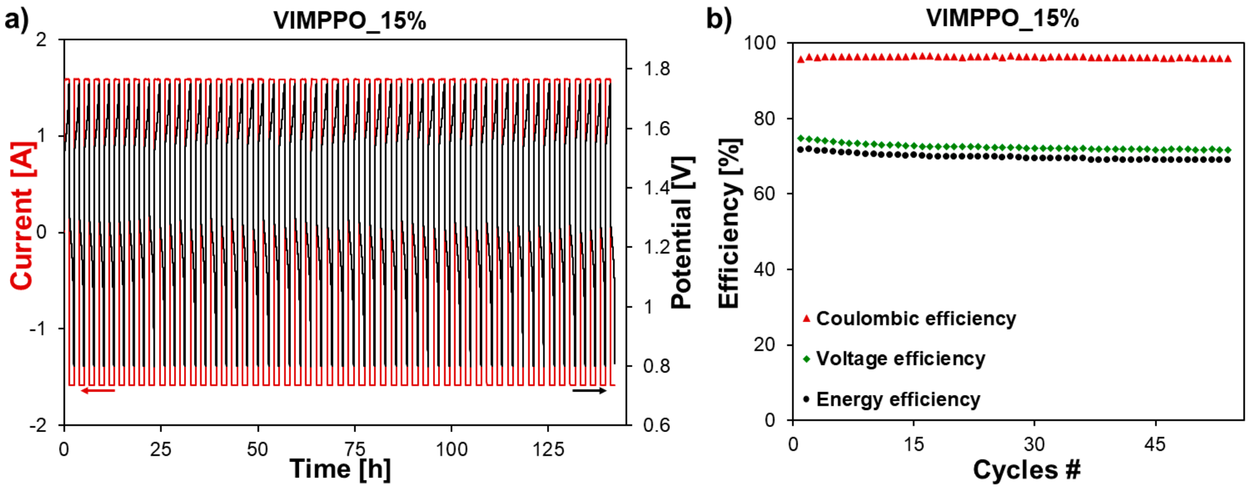

Figure S6. Cycling performance of the VRFB cell assembled with the membrane VIMPPO_15% - over 50 cycles at 80 mA cm^{-2}: a) charge discharge curves recorded, b) coulombic, voltage and energy efficiency of the cell.

Table S1. Performance of VRFB single cells (active area – 20 cm^2) assembled with different membranes.

Current Density [mA.cm^2]	VIMPPO_20%					
	Coulombic Efficiency$_{average}$ [%]	Standard Deviation	Voltage Efficiency$_{average}$ [%]	Standard Deviation	Energy Efficiency$_{average}$ [%]	Standard Deviation
20	93.3	0.6	91.7	0.6	85.5	0.4
50	97.1	0.3	79.1	1.2	76.8	1.1
80	97.9	0.3	68.5	1.0	67.1	0.9
	VIMPPO_15%					
20	91.5	1.2	93.7	0.9	84.7	3.2
50	95.8	1.0	85.0	1.4	81.5	2.2
80	97.9	0.8	76.7	0.9	75.1	1.0
	VIMPPO_10%					
20	84.3	1.6	92.5	0.3	78.0	1.7
50	93.1	1.0	82.7	0.8	77.0	1.4
80	95.2	0.8	73.8	1.0	70.0	1.6
	FAP 450					
20	95.4	0.9	92.5	0.3	88.2	0.7
50	97.9	0.4	82.5	0.3	80.8	0.3
80	98.3	0.7	74.3	0.4	73.0	0.6
	N115					
20	96.4	0.3	92.9	0.8	89.9	1.0
50	98.1	0.6	83.7	1.4	82.7	1.4
80	98.5	0.3	76.4	1.2	75.0	0.9

Table S2. Permeability coefficients calculated for the composite membranes and the commercial AEM – FAP 450.

	**Permeability Coefficients [cm^2 min^{-1}]**
VIM PPO 100%	1.34E-07
VIM PPO 25%	2.17E-07
FAP 450	4.82E-07
VIM PPO 20%	2.18E-07
VIM PPO 17%	3.42E-07
VIM PPO 15%	3.20E-07
VIM PPO 12%	3.73E-07
VIM PPO 10%	5.70E-07

In the case of the composite membranes the thickness of the entire membrane consists of the thickness of the porous support and the thickness of the layer. Since the permeation of vanadium ions is in majority slow down by the coating layer, the permeability coefficients were calculated taking the coating layer thickness into the consideration. This allows to compare the obtained results with the one of a dense FAP 450 membrane.
